# Production of New Biopesticides from *Cymbopogon citratus* for the Control of Coffee Rust (*Hemileia vastatrix*) under Laboratory and Field Conditions

**DOI:** 10.3390/plants12051166

**Published:** 2023-03-03

**Authors:** Luis Morales-Aranibar, Francisca Elena Yucra Yucra, Nivia Marisol Pilares Estrada, Policarpo Quispe Flores, Renatto Nicolino Motta Zevallos, Juan Carlos Llanos Zegarra, Uriel Palomino Trujillo, Carlos Genaro Morales Aranibar, Hebert Hernán Soto Gonzales, Jorge González Aguilera, Leandris Argentel-Martínez, Sucleidi Nápoles Vinent

**Affiliations:** 1 National Intercultural University of Quillabamba, La Convenciòn, Cusco 08741, Peru; 2 Jorge Basadre Grohmann National University (UNJBG), Tacna 23001, Peru; 3 National University of Moquegua (UNAM), Ilo 18601, Peru; 4 State University of Mato Grosso do Sul (UEMS), Cassilândia 79540-000, MS, Brazil; 5 Tecnológico Nacional de México/Instituto Tecnológico del Valle del Yaqui, Bácum 85270, Sonora, Mexico; 6 Universidad de Oriente, Santiago de Cuba 90600, Cuba

**Keywords:** *Coffee arabica*, lemon verbena, sustainable control methods, uredospores

## Abstract

One of the diseases with the greatest economic impact on coffee cultivation worldwide and particularly in Peru is coffee rust (*Hemileia vastatrix*). The search for sustainable control methods as disease management strategies in coffee cultivation is necessary. The objective of this research was to determine the effectiveness of five biopesticides based on lemon verbena (*Cymbopogon citratus*) for the control of rust applied in laboratory and field conditions to allow the recovery of coffee (*Coffea arabica* L. var. typica) in La Convención, Cusco, Peru. Five biopesticides (oil, macerate, infusion, hydrolate and Biol) and four concentrations (0, 15, 20 and 25%) were evaluated. The biopesticides were evaluated under laboratory conditions (light and dark) at different concentrations. The design used was completely randomized in a factorial scheme. The biopesticides were incorporated into the culture medium and inoculated with 400 uredospores of rust, and the germination percentage was evaluated. Under field conditions, the biopesticides at the same concentrations were evaluated for 4 weeks after application. Under these field conditions, the incidence, severity and area under the disease progress curve (AUDPC) of selected plants with a natural degree of infection were evaluated. In the laboratory, the results showed that all biopesticides were effective in reducing the germination of rust uredospores to values <1% of germination in relation to the control, which showed values of 61% and 75% in the light and dark, respectively, independent of the concentration used, with no significant differences between them. In the field, 25% oil promoted the best response with values <1% and 0% incidence and severity, respectively, in the first two weeks after application. The AUDPC for this same treatment showed values of 7 in relation to 1595 of the control. *Cymbopogon citratus* oil is an excellent biopesticide that can be used to control coffee rust.

## 1. Introduction

The disease that has had the greatest impact on coffee (*Coffee arabica*) cultivation worldwide and particularly in Peru is rust (*Hemileia vastatrix*), which has generated large economic losses [1]. This disease has made it necessary to rotate and substitute varieties, look for genotypes and cultivars with greater resistance, such as the Borbón coffee varieties [2]. Borbón coffee produces 30% more than *Coffea arabica* L. var. typica, which is highly susceptible to rust, and the size of its grains is smaller [3]. As a culture management strategy, many Quillabambino coffee growers in Peru have substituted their *C. arabica* L. var. typica coffee plantations with other rust-resistant coffees, such as Catimor [4].

Rust decline is also reduced with the use of biological control and cultural tillage [5]. However, several alternatives used for rust control include synthetic fungicides [6,7]. The use of large amounts of chemical fungicides damages the environment and generates an increase in crop production costs [8]. In general, the use of chemical fungicides should be used only in cases of phytosanitary emergencies, when rust is difficult to control [9], as has occurred in Quillabamba, where there has been the elimination of large extensions of *Coffea arabica* L. var. typica.

In Peru, coffee production was most affected by rust in 2013, with losses reaching 35% of production [10,11]. It is estimated that rust will cause losses of 60% of the crop in Peru, representing a value of more than 260 billion dollars [1]. These losses make it necessary to develop strategies to establish new scientifically, technically and economically based alternatives for the recovery of the coffee crop.

Among these new rust control strategies is the use of essential oils and aromatic volatile products, which are part of the secondary metabolites produced by plants. These compounds have already been studied and used in food [12,13], medicine [13] and agriculture industries for the control of fungi, bacteria and pathogens that attack plants [12,14,15,16,17]. Recent work has shown that this range of plant-derived products can constitute new alternatives as biofungicides for rust control [7].

The use of plants, or some of them, as biopesticides consists of a mixture of biomolecules with antifungal properties derived from the secondary metabolism of plants [18,19,20]. Currently, the application of products from plants is an effective alternative to synthetic pesticides, which are less expensive and biodegradable products [21,22].

*Cymbopogon citratus* is a herb of the Poaceae family (Gramineae) native to Southeast Asia [23]. This herb is distributed in various parts of the world, mainly in tropical regions and savannas, known by the common name of grass Luisa, “caña santa” or herbs calentura [24]. In humans, it is used as a decoction or infusion as an anticatarrhal, antispasmodic, hypotensive, antipyretic and tranquilizer [25,26]. Other research on the essential oils it produces has shown the antibacterial, antifungal, antiviral, insecticidal and antioxidant properties of lemon verbena [27,28,29]. Chemical studies of *Cymbopogon citratus* have shown antioxidant [30,31], anti-inflammatory and antifungal [32,33], inhibition of *Staphylococcus aureus* and *Escherichia coli* [34], larvicidal, ovicidal and is repellent against the filarial mosquito, *Culex quinquefasciatus* [35], among other applications.

The lack of information on biopesticides creates a current need for research on the generation of new alternatives in the control of rust to expand knowledge and help better understand these new forms of rust control, with less impact on the environment and our health. The goal of this study was to determine the efficacy of five biopesticides based on herbs Luisa for the control of rust applied in laboratory and field conditions, that allow the recovery of coffee (*Coffea arabica* L. var. typica) in La Convención, Cusco, Peru.

## 2. Results

### 2.1. Chemical Composition of the Biopesticides Employed

**Figure 1:**
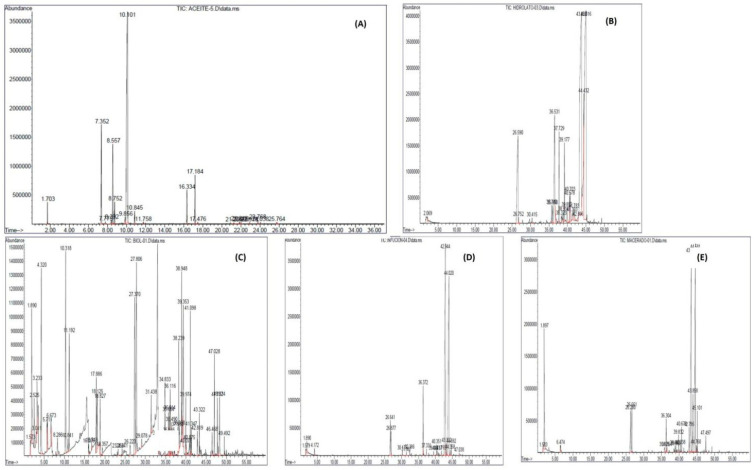
Results of the chemical composition using gas chromatography coupled to mass spectrometry of the biopesticides obtained from *Cymbopogon citratus* to be used in the control of coffee rust (**A**) oil, (**B**) biol, (**C**) hydrolate, (**D**) infusion, and (**E**) macerate.

As part of the characterization process of the biopesticides used for the control of coffee rust, the chemical characterization of each one was carried out, and the concentrations of the compounds obtained are described in Figure 1. A wide variety of chemical compounds was detected if we consider the total of them, some of which were present in only a few of them and others of which were common to all of them (Figure 1).

In the analysis of the oil, 24 active principles were identified (Figure 1A), and those that predominate in concentration are D-limonene (64.07%), α-citral (10%), α-pinene (9.83%), β-pinene (8.52%), γ-terpinene (1.29%), β-myrcene (2.04%) and β-citral (8.53%).

Hydrolate analysis shows 12 active principles (Figure 1B). Among the main ones found in higher concentrations are Photocitral A (2.48%), Linalool (7.98%), Sulcatone (8.83%), α-citral (33.28%) and β-citral (36.15%).

The biopesticides Biol analysis showed the highest number of compounds with 33 active principles (Figure 1C); those with the highest concentrations were 1-butanol (7.37%), acetic acid, butyl ester (3.63%), ethyl ester (1.63%), butanoic acid, ethyl ester (7.22%), butanoic acid, propyl ester (2.54%), hexanoic acid, butyl ester (2.76%), butyl butyrate (7.65%), ethyl butyrate (7.65%), ethyl caprylate, ethyl caproate, ethyl caproate (2.76%), hexanoic acid, butyl ester (2.76%), butyl butyrate (7.65%), ethyl caprylate, ethyl caproate, ethyl hexanoate (8.61%), ethyl cyclohexanoate (2.3%), cis-tagetone (3.99%), diethyl phthalate (neoxanthin, solvanol (2.27%), ethanol (5.43%) and p-cresol, methyl phenol (2.55%).

Analysis of the infusion showed 14 active principles (Figure 1D), the ones that stood out for their concentration were (R)-(+)- β-citronellol (42.32%), sulcatone (2.6%), α-citral (0.31%) and β-myrcene (3.3%).

The macerated analysis showed 10 active principles (Figure 1E), and those showing Citral (34.72%), ethanol (12.32%), geraniol (4.27%), and sulphur (4.27%) had the highest concentrations.

Most of the biopesticides used showed chemical stability after being processed, with the only exception being the infusion treatment, which we recommend using 24 h after processing, because after that time it decomposes and promotes bad odors.

### 2.2. Laboratory and Field Statistical Analysis

Upon verifying the ANOVA results (Table 1), it was observed that under field conditions, the factors evaluated (biopesticide, concentration and evaluation) showed highly significant triple interactions (*p* < 0.01), which indicates that there is a variable effect when combining the five biopesticides at the four concentrations evaluated during a period of four weeks under field conditions. Under these conditions, the coefficients of variation were low, showing the precision of the data obtained for the variable’s incidence, severity and AUDPC of coffee rust (Table 1).

When the ANOVA result was observed for the variables evaluated in the laboratory conditions (Table 1), a significant effect of the concentrations evaluated was barely observed (*p* < 0.01). For these two laboratory conditions (light and dark), the biopesticides evaluated did not differ in the germination variable independent of the light condition evaluated. For these conditions, moderate coefficients of variation were observed (Table 1).

### 2.3. Spore Germination Behavior under Laboratory Conditions

**Figure 2:**
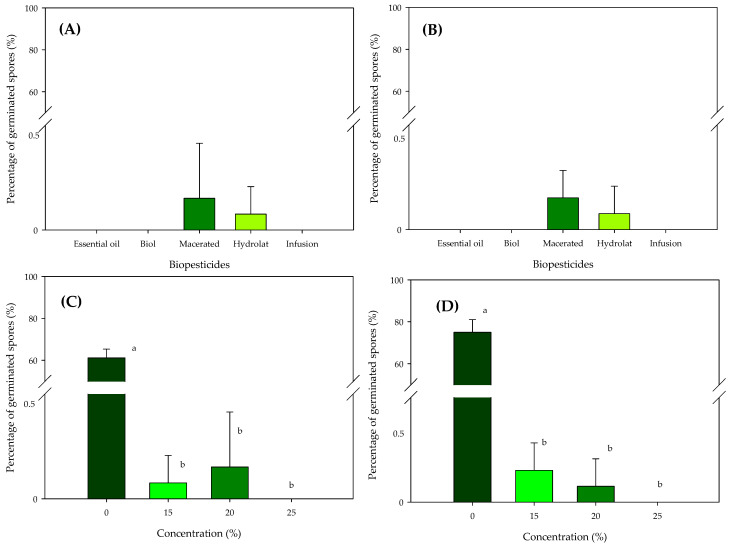
Comparison of means in relation to the percentage germination of rust uredospores in five biopesticides (**A**,**B**), applied at four concentrations (**C**,**D**) and evaluated under light (**A**,**C**) and dark (**B**,**D**) conditions in laboratory conditions. Different lowercase letters in the bars represent significant differences using Tukey’s test at 5% probability.

Observing the results of the percentage of rust uredospore germination under light (Figure 2A) and dark (Figure 2B) conditions indicates that there were no differences between the five biopesticides applied in relation to germination, independent of the condition evaluated (light or dark). For all the biopesticides, the germination percentages were extremely low (<1%), which shows their effectiveness, although the oil-based preparation, Biol and the infusion for the two conditions evaluated showed 0% germination out of a total of 400 uredospores which were placed on each plate for each of the replicates evaluated.

When considering the different concentrations used in our experiment, it was observed that regardless of the lighting condition (Figure 2C) or darkness (Figure 2D), there was a differentiation of the doses in relation to the control (zero biopesticide), which shows that the doses used were effective in preventing the germination of rust uredospores with values that were less than 1%, without showing differences between them in the two conditions tested (lighting and darkness). Only the 25% concentration showed control or inhibition of rust uredospore germination in 100% of them, independent of the illumination condition (Figure 2C,D).

### 2.4. Performance under Field Conditions

When the incidence variable is considered, the unfolding of the three-way interaction obtained is shown in Table 2 and Table 3. Table 3 shows the evaluation of the splitting of the biopesticides within the concentration levels in the four weeks. The results show that in the first and second weeks of evaluation, the biopesticides showed the same behavior when the concentrations were considered, obtaining the highest values of disease incidence at zero concentration, and as the concentration increased, the result obtained for this variable was inversely proportional, with the lowest values obtained at the 25% concentration of the biopesticide being independent of the type of biopesticide used, which shows that at this time of evaluation, all were efficient in reducing the incidence of the disease. When considering the doses in the first two weeks, the results show that regardless of the biopesticide, the zero dose (control) maintained the disease incidence constant. For the 15, 20 and 25% doses, the best treatment was the essential oil treatment with 39.7, 30.7 and 26.7% disease incidence, respectively, in the first week and 10.4, 5.3 and 1.0% incidence, respectively, in the second week, differing statistically from the rest of the treatments used.

**Figure 3:**
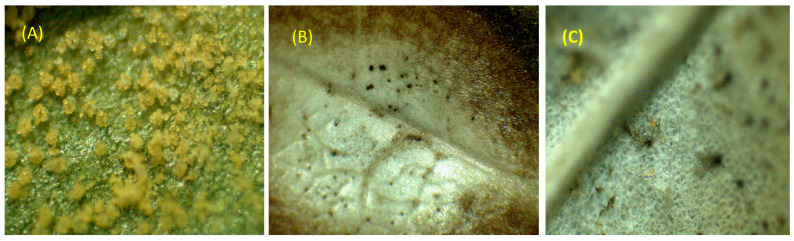
Detail of the evolution of the control of uredospores deposited on the leaves after application or treatment with oil. Image of the control without biopesticides (**A**), 15% oil after 24 h apparently the uredospores have burned (**B**) and after one week there is evidence of regeneration and disappearance of the rusts in the plant tissue (**C**).

The results show that for the third and fourth weeks of the evaluation, the biopesticides showed the same behavior when the concentrations were considered, obtaining similar behavior for the Biol, Macerated, Hydrolat and Infusion pesticides with the highest incidence values of the disease at zero concentration, and as the concentration increased, the incidence values decreased, with the lowest values obtained in the 25% concentration of the biopesticide, independent of the type of biopesticide used. For the essential oil biopesticide, the highest incidence was obtained in the control group (zero dose), differing from the rest of the concentrations, which did not differ from each other in the Tukey test at 5% probability (Figure 3).

When considering the different concentrations of biopesticides for the last two weeks, the results were similar to the first two weeks and showed that only in the zero dose (control) was the incidence of the disease high, with values above 89% (III weeks) and 95% (IV weeks), independent of the biopesticide used. For the 15, 20 and 25% doses, the best treatment was that of essential oil, confirming the results of the first two weeks, with a control percentage higher than 1.0, 0.7 and 0.2% of disease incidence, respectively, in the third week and 0.0% of incidence in the fourth week, significantly different from the rest of the treatments used, with the only exception of the last week in the 25% concentration, where this biopesticide was only slightly different from the treatment with macerate (Table 2).

In Table 3, even in the comparisons of the variable incidence, it is observed that for the control treatment (zero dose of biopesticide), the progression of the disease was maintained with values above 81% already in the first week until the last week evaluated, showing the aggressiveness that under natural conditions the disease had in the field. When the other concentrations of each of the biopesticides were observed, it was observed that all of them were able to reduce the advance of the disease, with the best control established at the highest dose of 25%, independent of the biopesticides applied. The best control was observed when using the essential oil, which promoted incidence values close to zero from week III for doses of 20 and 25% (Table 3). The 25% concentration was also able to promote control below 1% incidence in week IV for most of the biopesticides, with the exception of Biol, which, even at the highest dose, maintained an incidence of 5.7%.

When observing the results for the severity variable described in Table 4, a behavior similar to that of the incidence discussed above (Table 2) is observed. In the four weeks of evaluation of the disease under field conditions, it is observed that the highest dose (25%) independent of the five biopesticides used is the one that promotes the lowest severity values, with the lowest value obtained when using 25% of essential oil-promoting values below 2% of severity.

When considering the individual doses and the comparison between the different biopesticides, it was observed that for the zero dose (absence of biopesticides), the severity values were high (from 55.6 to 80%). For the other doses, with the exception of week IV for a dose of 25%, where the biopesticides did not differ, for the other evaluations, the essential oil was the one that stood out and differed from the rest of the treatments independent of the dose (15, 20 and 25%), showing the superiority of this treatment in relation to the others used.

Even when comparing the severity variable within each of the concentrations used, the data are shown in Table 5. For this comparison, it is observed that in the zero concentration (control) in the absence of biopesticides, the severity of the disease increases with the passing of the weeks, contrary to the behavior that occurs for the concentrations 15, 20 and 25% of biopesticide where most of them promote the decrease of the disease (Table 5). For all concentrations, regardless of the biopesticide applied, control was observed with a decrease in the percentages of severity over time, with the lowest values always observed in week IV, regardless of the concentration and the biopesticide applied. Even in this same comparison, the biopesticide based on essential oil was observed as the one that exerted the best control, promoting the lowest values regardless of the concentration applied.

**Figure 4:**
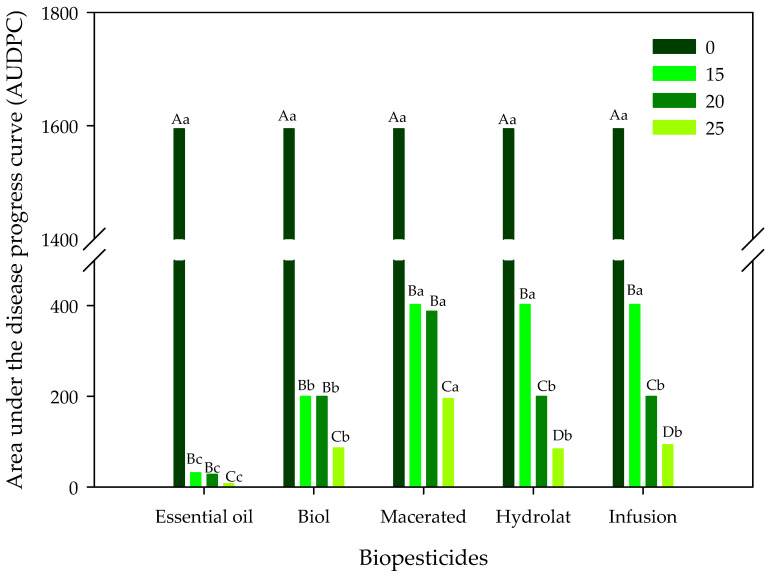
Comparison of means in relation to the area under the disease progress curve (AUDPC) when five biopesticides were applied at four concentrations. Uppercase letters for the comparison of doses and lowercase letters for the comparison of biopesticides represent significant differences using Tukey’s test at 5% probability.

**Figure 5:**
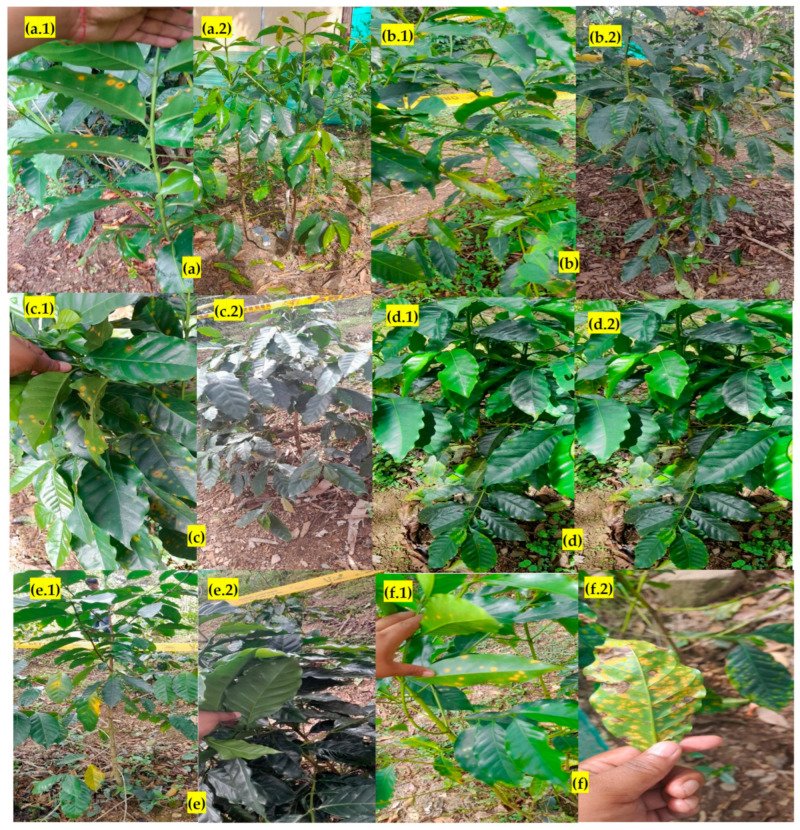
Biopesticide treatments before and after, (**a**) Oil (**b**) Infusion (**c**) Biol (**d**) Hydrolate (**e**) Macerated, (**f**) Control, (**a.1**,**b.1**,**c.1**,**d.1**,**e.1**,**f.1**) detail of the coffee plants without treatments, (**a.2**,**b.2**,**c.2**,**d.2**,**e.2**) fourth week after applying the different treatments, the recovered coffee plants are observed, except in the control (**f2**).

For the variable AUDPC, significant interactions were detected using ANOVA, and the comparison of the means of the treatments is shown in Figure 4. Regardless of the biopesticide applied, all the doses (15, 20 and 25%) differed from the control (zero dose with the highest value of 1595); on the other hand, the highest concentration (25%) promoted the lowest values regardless of the biopesticide applied (Figure 4). When the biopesticides were compared within each of the doses applied (Figure 5), it was observed that in the zero dose they did not differ, while in the other three doses applied, the essential oil biopesticide was always the one that stood out with the lowest AUDPC values, differing statistically from the other treatments used.

## 3. Discussion

Different studies have shown the effectiveness of plant-derived preparations in control of different phytopathogenic fungi causing damage to agricultural crops. To date, these studies have mainly focused on inhibiting growth under in vitro conditions, and few studies have been tested in the field [36,37]. Coffee rust is one of the main phytopathogenic fungi of worldwide importance due to the economic damage it causes and for which the search for control alternatives is necessary [4,6,7,10,38]. As part of these alternatives, the present work shows that control with biopesticides based on *Cymbopogon citratus* is possible, thus generating a new alternative for field control of coffee rust.

When we evaluated the composition of the different preparations obtained from the same plant, a wide range of active principles was found when characterizing the five biopesticides made from *Cymbopogon citratus* (Figure 1). These results show that this plant, regardless of how we use it (macerate, hydrolysate, oil, infusion, among other forms of use), has a wide range of products and metabolites that can exert control on diverse pathogens and hence its importance in its use as a source or raw material of bioproducts for the control, mainly of fungi, as obtained in the present work. The characterization results do not encourage the development of new strategies for the control of other fungi or pathogens if the active principles present in these biopesticides are considered, which lays the foundation for further studies. It has already been described that *Cymbopogon citratus* has a broad spectrum of antifungal activities in vitro thanks to the high content of α-Citral that inhibits mycelial growth and spore germination in *Botrytis cinerea* [17]. This substance, α-Citral, was detected in the oil (10%), hydrolysate (33.28%) and infusion (0.31%) elaborated in the present work.

Plants in general are capable of producing a wide range of products that allow us at any time to describe new formulations and obtain new results. Similarly, Plaus et al. [39], working with *Origanum vulgare* oil, demonstrated that terpineols, phenols and compounds metabolically related to carvacrol, had an antimicrobial activity with contents of carvacrol 9%, terpineol 12.19%, and P-cymene 6.86%.

The results under laboratory conditions showed a more accentuated inhibition of the growth of rust uredospores (Figure 2) compared to the field results. In these controlled conditions, germination percentages were lower than 1% for the five biopesticides evaluated, and of these, oil, Biol and infusion showed values of 0% germination (Figure 2). These results were obtained independently of the illumination condition tested. Under these same conditions, the different concentrations of the biopesticides tested were effective in inhibiting the germination of rust uredospores, although they did not differ between them in the two conditions tested (Figure 2). The 25% concentration showed 0% germination of rust uredospores independent of the illumination condition (Figure 2C,D). When we scale up the same biopesticides to the field, we observed that the control of the main variables that we controlled in the laboratory was lost, and with that, the high effectiveness becomes more pronounced for the oil that stands out from the rest. It is possible that the stability of the oil when applied in the field confirms other studies based on essential oils used in food [12,13,17], medicine [13] and agriculture in the control of pathogenic fungi and bacteria that attack plants and fruits [12,14,15,16,17].

The results obtained in the field show that among the five biopesticides tested, lemon verbena oil (*Cymbopogon citratus*) was the best treatment for rust control, promoting low values of incidence (at the IV week with 25% concentration values of 0%, Table 3) and severity (at the II week with 25% concentration values of 0%, Table 5) in field conditions compared to the control. The concentration of 25% used can be considered high, although its control effect was verified. Future work can define lower concentrations that continue to maintain the effectiveness of the oil in controlling this pathogen. From a practical point of view, the use of this concentration is possible, but reducing it increases the possibilities of use, reducing the cost of obtaining this recipe, if the effectiveness of the product is maintained.

Similar results on the use of essential oils for fungal growth inhibition were reported by Chang et al. [40]. The authors comment that plant oils can inhibit mycelial growth and conidial germination, hyphal mass, germ tube, promote cell wall and membrane rupture as some of the benefits of extradosed essential oils from some plants. In other studies, the oils of other plants, such as lemon, orange and bergamot, have been shown to be effective in the microbiological control of some pathogens, evidencing the presence of main active ingredients such as limonene (72.5 and 76.4%) and β-pinene (11.6–18.7%) [12]. Marei et al. [15] demonstrated that thymol, (S)-limonene and 1,8-cineole have effective antifungal action by inhibiting *Rhizoctonia solani, Fusarium oxysporum, Penecillium digitatum* and *Asperigallus niger* in their studies. Guédeza et al. [16] demonstrated the antifungal action of limonene oils against *Colletotrichum gloeosporioides*, *Penicillium indicum*, *Fusarium solani*, *Rhizopus stolonifer* and *Aspergillus flavus*.

Yan et al. [41], using essential oils of *Cymbopogon citratus*, *Thymus vulgraris* and *Origanum heracleoticum*, confirmed within their active principles α-citral (38.34%), β-citral (29.51%), thymol (22.71%), p-cymene (20.43%), γ-terpinene (11.47%) and carvacrol (37.47%), which are responsible for damage to the plasma membrane, evidencing losses of intercellular sugars, proteins and nucleic acids. Wu et al. [42], in studies of juniper essential oil, showed in vitro antifungal activity against the fungus *B. cinerea*, confirmed with scanning electron microscopy that the mycelia were wrinkled, twisted and distorted. The active components of the product evaluated by the authors were limonene (15.17%), γ-terpinene (8.3%), β-myrcene (4.56%), terpinene-4-ol (24.26%), linalool (8.73%), α-terpineol (1.03%), and o-cymene (8.35%). A similar study was developed by EL Abdali et al. [43], who investigated the antioxidant, antifungal and insecticidal activities of the essential oil extracted from *Lavandula dentata*, verifying that the active principles found were linalool (45.06%), camphor (15.62%) and borneol (8.28%), which inhibited the mycelial growth of *B. cinerea, A. alternata* and *F. oxysporum* in vitro. Many of these active principles were found in the five tested biopesticides based on *Cymbopogon citratus*, which justifies the results obtained.

We presume that our study of the elaboration of biopesticides based on *Cymbopogon citratus* could be an alternative for the control of rust (*Hemilea vastartix*) since the active compounds present in each of the biopesticides have active principles that other authors have determined and used for the control of different pathogenic fungi. In our study, the oil of *Cymbopogon citratus* had 64.07% D-limonene as the main component of the oil, in addition to having 10% α-citral, 9.83% α-pinene, 8.53% β-citral, and 8.52% β-pinene, which would be the main components responsible for rust inhibition in the field and laboratory.

The concentrations of 15, 20 and 25% showed effectiveness against rust under laboratory and field conditions. The concentrations used showed the same response of the biopesticides if we considered the laboratory and field, all of which were higher than the zero control and with values lower than 1% germination independent of the lighting conditions tested. When these same concentrations were evaluated in the field, the higher concentration, although it showed some leaf damage (necrosis of plant tissue), proved to be the best option in combination with the oil-based biopesticide. For future work, the field application conditions of the biopesticides should be adjusted to promote the least damage to the plant and the highest percentage of control to maintain the lethal dose of the oil-based rust of *Cymbopogon citratus*.

In the same way as the oil of *Cymbopogon citratus*, the results of the hydrolysate show a higher proportion of α-Citral (33.28%) and β-Citral (36.15%) in its composition, presuming that these active principles are fundamental in the control response obtained for rust. These two processes of obtaining oil and hydrolysate have a high cost for the farmer. For this reason, other derivatives of *Cymbopogon citratus* were tested, and maceration was proposed. The results show that the macerate had the highest amounts of citral (34.72%), β-citral (30.21%) and geraniol (4.27%), and although it was not the best control treatment for rust, the results demonstrate its effectiveness in the field and laboratory. Another biopesticide that was tested with a faster and lower cost process was the infusion based on *Cymbopogon citratus*. It was determined that its main active principles were (R)-(+)- β-citronellol (42.32%), geraniol (37.89%), linalool (5.95%) and β-myrcene (3.3%), which contribute to the control response obtained in the field and laboratory. In addition to the control response on rust, it was evidenced that in the first two weeks of having applied the infusion in all the different contractions tested, the coffee plants showed new shoots, a result that evidence other applications of this biopreparation that should be verified in future work.

## 4. Materials and Methods

### 4.1. Location of the Experimental Area in the Field

The experiments were established during the cycle from December 2021 to August 2022, the wet period, in the town of Huayanay, a coffee-producing area in Quillabamba, province of La Convención, Cusco-Peru. Huayanayay is located at UTM 18 L 755216 west longitude and 8582960 south latitude at an altitude of 1515 m above sea level. The region is characterized by crops of *Coffea arabica* L. var. typica, naturally contaminated with rust (*Hemilea vastartix*), within which the experimental plot was delimited with dimensions of 30 m by 21 m, with plants spaced 1.5 m apart and 1.70 m between rows. The average temperature of the region during the field experiment was 28.1 °C, and the relative humidity was 84.6%.

Soil analysis of the experimental plot was carried out by means of texture analysis using the hydrometer method, and as a result, a sandy clay loam soil with a pH of 5.18 was found.

Water analysis was performed, and the results showed electrical conductivity values of 0.05 mmhoscm, pH 4.45, total hardness 21.41 mg CaCO L3 ^−1^, SAR of 0.11, calcium 0.33 meq L^−1^, magnesium 0.1 meq L^−1^, and chlorides 0.11 meq L^−1^.

### 4.2. Field Experimental Design

The experimental design used was a randomized complete block with a 5 × 4 × 4 factorial arrangement, with three replicates per treatment. The sources of variation used were five types of biopesticides (essential oil, hydrolysate, macerate, infusion and biol, all prepared from lemon verbena (*Cymbopogon citratus*) at four different concentrations (0, 15, 20 and 25% *v*/*v*) and four evaluation moments (1, 2, 3 and 4 weeks after the application of the biopesticide).

### 4.3. Preparation of Biopesticides

*Cymbopogon citratus* essential oil and hydrolysate: Fresh leaves of *Cymbopogon citratus* were used as the main ingredient for processing. The leaves were processed using the steam entrainment method recommended by [44]. The oil and the aqueous liquid (hydrolate) were obtained directly. The hydrolysate was preserved in polypropylene bottles properly labelled for later use in a shaded environment at 27 °C and an average relative humidity of 55%, while the oil was preserved in an amber-colored bottle at 4 °C in the refrigerator to then concentrations were prepared according to the experimental design before use.

*Cymbopogon citratus* macerate: 6.8 L of 40% ethanol was used to make the macerate, 3.4 kg of *Cymbopogon citratus* was added, and the mixture was shaken manually and mechanically for 30 min [45]. Subsequently, it was left to settle in amber bottles under the penumbra for 30 days at 27 °C and an average relative humidity of 55%. Concentrations were prepared according to the experimental design before use.

Infusion of *Cymbopogon citratus*: For its preparation, a stainless steel pot was used in which 6.8 L of water was placed, and when it began to boil, 3.4 kg of *Cymbopogon citratus* was added. It was left to stand for 24 h and then filtered, discarding the solid residue [46]. The concentrations of the liquid extract obtained were prepared according to the experimental design before use.

Biol made with *Cymbopogon citratus*: For its production, a container containing 4 kg of fresh cattle manure was used, to which 200 g of ash, 20 g of granulated dry yeast, 0.2 L of molasses were added, and then 500 g of lemon verbena [47]. The entire mixture was placed in a 50 L container, where the final volume was completed to 20 L by adding a tap water and then allowed standing for 30 days in dark conditions at 27 °C and an average relative humidity of 55%. Subsequently, the concentrations were prepared according to the experimental design before use.

In all cases, the biopesticides prepared represent 100% concentration (pure), so dilutions were used for their application, and water was added to obtain concentrations of 15%, 20% and 25% for each biopesticide for use in the laboratory and field.

### 4.4. Chromatographic Analysis of Biopesticides

A sample of all biopesticides was used to analyze the chemical composition using gas chromatography coupled to spectrometry.

The chemical composition of the oil was determined using an Agilent Technologies 7890 Gas Chromatograph with an Agilent Technologies 5975C mass spectrometer detector with a J&W 122-1545.67659 DB-5ms Column, at 325 °C and 60 m × 250 µm × 0.25 µm. Temperature ramp with start cycle at 40 °C and ramp up at 5 °C per min to 180 °C, then ramp up at 1 °C per min to 200 °C for 2 min and finally 25 °C per min to 300 °C held for 3 min, for a run time of 45 min. The injection volume was 1 µL and a split of 30:1, with He carrier gas 1 mL min^−1^, for which 20 µL of the sample was taken and diluted in 1 mL of dichloromethane, to inject 1 µL of the solution to the equipment.

To determine the chemical composition of the Hydrolate, Macerated, Infusion and Biol this was performed using the Agilent 6890 N Gas Chromatograph, with Agilent 5975B mass detector, with Agilent HP-5MS 5% Phenyl Methyl Siloxane Column 30 m × 0.25id × 0.5 µm film, initial oven temperature 40 °C for 5 min, 1.5 °C increment per minute up to 80 °C, 5 °C per minute up to 200 °C, 1 min at 200 °C. The analysis time was 59.67 min, a splitless mode injection port was used, and the initial temperature was 250 °C. The type of gas used was helium, and its flow rate was 1.0 mL min^−1^ with SPME injection.

### 4.5. Laboratory Tests

Potato + dextrose + agar (PDA) medium was prepared in Erlenmeyer flasks and autoclaved; subsequently, the corresponding dose of each biopesticide (0, 15, 20 and 25%) was added to the medium. Once the medium solidified, 400 uredospores were seeded [48]. Uredospores from infested leaves were employed. Directly with the help of a scalpel, the uredospores were placed in Petri dishes containing distilled water, and then uredospores were counted in a Newbauer chamber. For the experiment, 400 uredospores were counted and incubated in each of the plates with the different treatments, including control without biopesticide.

Germination of uredospores was evaluated under light conditions (%). For this purpose, the inoculated plates were incubated at 28 °C for 6 h under direct natural light conditions. After that time, the germinated uredospores were counted under a microscope. Similarly, germination under dark conditions (%) was performed, for which the Petri dishes were covered with kraft paper and incubated at 28 °C for 6 h. Then, the germinated uredospores were counted with the help of a microscope.

The average laboratory temperature was 28 °C, and the relative humidity was 55.9%.

### 4.6. Field Trials

As a first step, the incidence and severity of the plants that will constitute the plots were evaluated as described by SENASA [49] before starting the application of the different treatments. Within the experimental plots, an incidence of over 80% and severity of 55.6 were verified prior to the application of the treatments as an average. Three replicates with three plants each and the same infestation pattern were marked. The evaluation of incidence and severity was carried out during the four weeks of the experiment.

The different biopesticides were applied in the afternoon between 15 and 16 pm using a 15 L manual sprayer. This time was selected because the environmental conditions were the mildest in it, facilitating the non-evaporation of the product. The application was carried out during the four weeks, evaluating the incidence and severity each week [48]. After each application of the treatments, the sprayer was washed to avoid contamination or mixing of treatments.

With the severity records, the area under the disease progress curve (AUDPC) was determined using the trapezoidal integration method described by Campbell and Madden [50] using the equation:

$$\mathrm{AUDPC}=\sum_{i=1}^{n-1}\left[\frac{X_{i+1}+X_{i}}{2}\right]\left(t_{i+1}-t_{i}\right)$$

where:

*x_i_* = percentage of severity in each evaluation, *t* = time (days) and *n* = number of evaluations.

### 4.7. Statistical Analysis

The experimental data were tested to verify the assumptions of normality and homogeneity. Subsequently, the data were subjected to a pooled analysis of variance (ANOVA), and when significant, the means were compared using Tukey’s test at 5% probability. Regression analysis was employed to evaluate the effect of biopesticide doses, and significant equations with the highest coefficients of determination were fitted (F test, *p* < 0.05). Statistical analyses were performed with Rbio software [51] and SigmaPlot 10.0^®^ (Systat Software, Inc., San Jose, CA, USA.) was used for plotting.

## 5. Conclusions

All bioplaguicides based on *Cymbopogon citratus* described contain a wide range of active principles capable of controlling coffee rust (*Hemileia vastatrix*). Under laboratory conditions, bioplaguicides were tested and the efficiency of the rust control was determined, with inhibition of germination at values below 1%, demonstrating the effectiveness of all of them. In the field, the control of the rust was effective when the oil was used regardless of the concentration (15, 20 and 25%), controlling the disease at 0% of severity and intensity after application. We recommend the 15% acceptance rate as the best treatment. The results obtained show the potential of *Cymbopogon citratus* and its derivatives (treatments used) in the control of coffee beans as an alternative and effective method in relation to chemical control.

## Figures and Tables

**Table 1:** ANOVA sum of squares results obtained when comparing five biopesticides, applied at four concentrations and evaluated on four different dates on coffee crop for rust control.

FV ^1^	GL ^2^	Field			GL	Laboratory	
		Incidence (%)	Severity (%)	AUDPC ^3^		GLight (%)	GDark (%)
Biopesticides (B)	4	3338 ***	854 ***	5 ***	4	3	3
Concentration (C)	3	56582 ***	62370 ***	4 ***	3	4 ***	5 ***
Evaluation (E)	3	15929 ***	1010 ***				
B × C	12	485 ***	191 ***	2 ***	12	2	2
B × E	12	329 ***	142 ***				
C × E	9	3381 ***	1709 ***				
B × C × E	36	48 ***	30 ***				
Waste	160	0.37	0.27	3	40	5	4
CV (%)		1.39	1.99	1.31		26.75	16.03

^1^ FV: sources of variation, CV: coefficient of variation. ^2^ GL: degrees of freedom. ^3^ AUDPC: area under the disease progress curve. GLight: germination under light conditions, GDark: germination under dark conditions. *** represents statistical significance at 0.01% probability for the ANOVA F test.

**Table 2:** Comparison of means in relation to the incidence of five biopesticides on coffee crops for rust control, applied at four concentrations and evaluated on four different dates after field application.

Biopesticides/Concentration	I Week				II Week			
	0	15	20	25	0	15	20	25
Essential oil	81.7 Aa	39.7 Bc	30.7 Cd	26.7 Of	86.77 Aa	10.4 Be	5.3 Cd	1.0 Of
Biol	81.7 Aa	78.3 Ba	67.9 Cb	49.2 Dc	86.77 Aa	63.4 Ba	55.3 Ca	35.4 Da
Macerated	81.7 Aa	79.1 Ba	69.2 Cab	53.3 Db	86.77 Aa	49.0 Bd	38.9 Cc	28.7 Dc
Hydrolat	81.7 Aa	79.6 Ba	69.8 Ca	54.9 Da	86.77 Aa	57.0 Bb	45.6 Cb	30.3 Db
Infusion	81.7 Aa	75.5 Bb	58.0 Cc	45.7 Dd	86.77 Aa	55.4 Bc	45.4 Cb	21.6 Dd
Biopesticides/Concentration	III Week				IV Week			
	0	15	20	25	0	15	20	25
Essential oil	89.73 Aa	1.0 Bd	0.7 Be	0.2 Bc	95.67 Aa	0.0 Bd	0.0 Bd	0.0 Bb
Biol	89.73 Aa	37.8 Ba	21.5 Ca	14.7 Da	95.67 Aa	19.5 Ba	9.8 Ca	5.7 Da
Macerated	89.73 Aa	22.7 Bc	18.0 Cc	7.8 Db	95.67 Aa	7.7 Bc	1.7 Cc	0.2 Db
Hydrolat	89.73 Aa	31.1 Bb	15.0 Cd	8.9 Db	95.67 Aa	9.0 Bc	4.4 Cb	0.7 Db
Infusion	89.73 Aa	31.2 Bb	19.9 Cb	8.3 Db	95.67 Aa	11.1 Bb	3.7 Cb	0.4 Db

Uppercase letters in the line and lowercase letters in the column represent significant differences using Tukey’s test at 5% probability.

**Table 3:** Comparison of means in relation to the incidence of five biopesticides in the coffee crop for the control of rust, applied at four concentrations and evaluated on four different dates after field application.

Biopesticides/Concentration	Concentration 0				Concentration 15			
	I Week	II Week	III Week	IV Week	I Week	II Week	III Week	IV Week
Essential oil	81.7 D	86.8 C	89.7 B	95.7 A	39.7 A	10.4 B	1.0 C	0.0 C
Biol	81.7 D	86.8 C	89.7 B	95.7 A	78.3 A	63.4 B	37.8 C	19.5 D
Macerated	81.7 D	86.8 C	89.7 B	95.7 A	79.1 A	49.0 B	22.7 C	7.7 D
Hydrolat	81.7 D	86.8 C	89.7 B	95.7 A	79.6 A	57.0 B	31.1 C	9.0 D
Infusion	81.7 D	86.8 C	89.7 B	95.7 A	75.5 A	55.4 B	31.2 C	11.1 D
Biopesticides/Concentration	Concentration 20				Concentration 25			
	I Week	II Week	III Week	IV Week	I Week	II Week	III Week	IV Week
Essential oil	30.7 A	5.3 B	0.7 C	0.0 C	26.7 A	1.0 B	0.2 B	0.0 B
Biol	67.9 A	55.3 B	21.5 C	9.8 D	49.2 A	35.4 B	14.7 C	5.7 D
Macerated	69.2 A	38.9 B	18.0 C	1.7 D	53.3 A	28.7 B	7.8 C	0.2 D
Hydrolat	69.8 A	45,6 B	15.0 C	4.4 D	54.9 A	30.3 B	8.9 C	0.7 D
Infusion	58.0 A	45.4 B	19.9 C	3.7 D	45.7 A	21.6 B	8.3 C	0.4 D

Different capital letters in the line represent significant differences using Tukey’s test at 5% probability.

**Table 4:** Comparison of means in relation to the severity of five biopesticides for the control of coffee rust, applied at four concentrations and evaluated on four different dates after field application.

Biopesticides/Concentration	I Week				II Week			
	0	15	20	25	0	15	20	25
Essential oil	55.6 Aa	5.0 Bc	4.0 Bc	2.0 Cc	80.0 Aa	2.0 Bc	2.0 Bc	0.0 Cc
Biol	55.6 Aa	25.0 Bb	25.0 Bb	10.0 Cb	80.0 Aa	10.0 Bb	10.0 Bb	5.0 Cb
Macerated	55.6 Aa	40 Ba	40.0 Ba	25.0 Ca	80.0 Aa	25.0 Ba	25.0 Ba	10.0 Ca
Hydrolat	55.6 Aa	40 Ba	25.0 Cb	10.0 Db	80.0 Aa	25.0 Ba	10.0 Cb	5.0 Db
Infusion	55.6 Aa	40 Ba	25.0 Cb	10.0 Db	80.0 Aa	25.0 Ba	10.0 Cb	5.0 Db
Biopesticides/Concentration	III Week				IV Week			
	0	15	20	25	0	15	20	25
Essential oil	80.0 Aa	0.0 Bc	0.0 Bc	0.0 Bc	80.0 Aa	0.0 Bc	0.0 Bc	0.0 Ba
Biol	80.0 Aa	5.0 Bb	5.0 Bb	2.0 Cb	80.0 Aa	2.0 Bb	2.0 Bb	0.7 Ca
Macerated	80.0 Aa	10.0 Ba	8.3 Ca	5.0 Da	80.0 Aa	5.0 Ba	4.0 Ba	0.7 Ca
Hydrolat	80.0 Aa	10.0 Ba	5.0 Cb	2.0 Db	80.0 Aa	5.0 Ba	2.0 Cb	0.0 Da
Infusion	80.0 Aa	10.0 Ba	5.0 Cb	3.0 Db	80.0 Aa	5.0 Ba	2.0 Cb	0.7 Da

Uppercase letters in the line and lowercase letters in the column represent significant differences using Tukey’s test at 5% probability.

**Table 5:** Comparison of means in relation to the severity of five biopesticides in coffee for the control of rust, applied at four concentrations and evaluated on four different dates after field application.

Biopesticides/Concentration	Concentration 0				Concentration 15			
	I Week	II Week	III Week	IV Week	I Week	II Week	III Week	IV Week
Essential oil	55.6 B	80.0 A	80.0 A	80.0 A	5.0 A	2.0 B	0.0 C	0.0 C
Biol	55.6 B	80.0 A	80.0 A	80.0 A	25.0 A	10.0 B	5.0 C	2.0 D
Macerated	55.6 B	80.0 A	80.0 A	80.0 A	40.0 A	25.0 B	10.0 C	5.0 D
Hydrolat	55.6 B	80.0 A	80.0 A	80.0 A	40.0 A	25.0 B	10.0 C	5.0 D
Infusion	55.6 B	80.0 A	80.0 A	80.0 A	40.0 A	25.0 B	10.0 C	5.0 D
Biopesticides/Concentration	Concentration 20				Concentration 25			
	I Week	II Week	III Week	IV Week	I Week	II Week	III Week	IV Week
Essential oil	4.0 A	2.0 B	0.0 C	0.0 C	2.0 A	0.0 B	0.0 B	0.0 B
Biol	25.0 A	10.0 B	5.0 C	2.0 D	10.0 A	5.0 B	2.0 C	0.7 D
Macerated	40.0 A	25.0 B	8.3 C	4.0 D	25.0 A	10.0 B	5.0 C	0.7 D
Hydrolat	25.0 A	10.0 B	5.0 C	2.0 D	10.0 A	5.0 B	2.0 C	0.0 D
Infusion	25.0 A	10.0 B	5.0 C	2.0 D	10.0 A	5.0 B	3.0 C	0.7 D

Different capital letters in the line represent significant differences using Tukey’s test at 5% probability.
